# Descemet Membrane Detachment Presenting as Graft Edema After Arcuate Keratotomy in an Eye with Previous Penetrating Keratoplasty—A Case Report

**DOI:** 10.3390/reports9030302

**Published:** 2026-09-10

**Authors:** Mohammed M. Abusayf, Nada F. Alsaif, Razan A. Alotaibi

**Affiliations:** 1 Department of Ophthalmology, College of Medicine, King Saud University, Riyadh 12372, Saudi Arabia; nadafaalsaif@gmail.com (N.F.A.);; 2 King Saud University Medical City, King Saud University, Riyadh 12372, Saudi Arabia; 3 Refractive Surgery and Myopia Research Group, Research Excellence Center in Ophthalmology and Visual Sciences, Department of Ophthalmology, College of Medicine, King Saud University, Riyadh 12372, Saudi Arabia

**Keywords:** Descemet membrane detachment, graft rejection, arcuate keratotomy, penetrating keratoplasty, corneal edema, rebubbling, anterior segment OCT, case report

## Abstract

**Introduction and Clinical Significance**: Descemet membrane detachment (DMD) is an uncommon but potentially vision-threatening complication of anterior segment surgery. Although immune-mediated graft rejection is a recognized cause of postoperative graft edema following arcuate keratotomy (AK) in eyes with previous penetrating keratoplasty (PKP), structural complications such as DMD may present with similar clinical findings and require fundamentally different management. We report a case of DMD initially misdiagnosed as acute graft rejection following manual AK in a post-PKP eye. **Case Presentation**: A 47-year-old female with a 20-year history of PKP in the right eye (RE) presented with decreased vision three weeks after undergoing manual AK using a diamond blade to correct high astigmatism. The procedure was complicated by an intraoperative wound leak requiring suturing. Postoperatively, she developed corneal edema and was initially misdiagnosed with acute graft rejection. Despite treatment with topical and systemic corticosteroids, her condition did not improve. Upon referral to our clinic, slit-lamp examination and anterior segment optical coherence tomography (AS-OCT) revealed a near-total, nonplanar DMD. The patient underwent a single air descemetopexy session involving three sequential intracameral air injections, resulting in Descemet membrane apposition and improvement in graft clarity. At 3-month follow-up, visual acuity had improved from hand motion to 20/200, with IOP within normal limits. Longer-term follow-up was unavailable. **Conclusions**: DMD should be considered as a cause of graft edema after AK in post keratoplasty eyes especially in the presence of complications.

## 1. Introduction and Clinical Significance

The Descemet membrane (DM) is the basement membrane produced by corneal endothelial cells. It plays a crucial role in maintaining corneal transparency. Significant damage to this layer often necessitates endothelial keratoplasty to restore corneal function [1,2]. The first documented case of Descemet membrane detachment (DMD) was reported by Weve in 1927 [2].

The incidence of DMD has been reported as 2.5% during or after extracapsular cataract extraction and between 0.044% and 0.5% during or after phacoemulsification. However, these rates may be underestimated, as DMD often occurs subclinically. Studies utilizing gonioscopy have reported a significantly higher incidence, with rates reaching up to 47% [3]. The most common cause of DMD is iatrogenic, particularly after intraocular surgeries. Cataract surgery is the procedure most frequently linked to DMD, especially following extracapsular cataract extraction and phacoemulsification, with incidences of 2.5% and 0.5%, respectively [3]. DMD has also been reported in several other surgical interventions, including keratoplasty, pars plana vitrectomy, iridectomy, trabeculectomy, laser capsulotomy, laser sclerostomy, and viscocanalostomy [3,4,5].

Persistent corneal edema following intraocular surgeries, including cataract extraction, keratoplasty, glaucoma procedures, and radial keratotomy, can signal the presence of DMD. Common clinical manifestations include delayed or insufficient visual recovery, a sensation of a foreign body in the eye, and excessive tearing. A slit-lamp examination typically reveals a localized corneal edema. The delineation of DMD margins can be assessed based on the extent of corneal haze; however, significant edema may obscure the characteristics of the detached membrane. In instances of localized DMD, spontaneous reattachment may occur, potentially leading to subsequent scarring of the membrane. Conversely, persistent corneal decompensation can culminate in compromised visual acuity and chronic discomfort due to the formation of bullae. Therefore, prompt management of DMD is imperative to mitigate corneal morbidity [1]. Prior to the development of Anterior Segment Optical Coherence Tomography (AS-OCT), the primary method for diagnosing diseases such as DMD relied on slit lamp examinations. However, significant corneal opacity can impede the timely identification of DMD. The introduction of AS-OCT has revolutionized this process by providing high-resolution images, which enhance the visualization of corneal anatomy [6].

High postoperative astigmatism remains one of the leading causes of suboptimal visual outcomes after otherwise successful Penetrating keratoplasty (PKP). When spectacles or contact lenses fail to provide satisfactory visual rehabilitation, surgical options such as arcuate keratotomy (AK) are commonly employed to reduce corneal astigmatism [7].

AK is a surgical technique that involves making one or two deep corneal incisions perpendicular to the steepest meridian of astigmatism. It is typically performed at least 3–4 months after all sutures have been removed to ensure corneal stability. Several incision patterns have been explored, including non-perforating, straight transverse, and AK, all of which aim to reduce corneal astigmatism [8].

Although AK is generally considered safe and effective, graft-related complications have been reported. Previous studies have documented episodes of immune-mediated graft rejection following both manual and femtosecond-assisted AK (FSAK), with reported incidences ranging from approximately 2% to 8%. Fortunately, most reported cases responded well to topical or systemic corticosteroid therapy [9,10,11,12].

To our knowledge, DMD has not been widely recognized as a potential cause of postoperative graft edema following AK in eyes with previous PKP. Failure to distinguish DMD from graft rejection may delay appropriate management because these entities require fundamentally different treatments.

We report a case with previous PKP who developed diffuse graft edema after complicated manual AK and was initially treated as acute graft rejection. AS-OCT subsequently demonstrated a near-total DMD, resulting in a change in diagnosis and management. This case highlights the importance of considering DMD in the differential diagnosis of postoperative graft edema following AK and emphasizes the value of early AS-OCT imaging.

## 2. Case Presentation

A 47-year-old woman with keratoconus had undergone PKP in the right eye (RE) 20 years earlier. Three weeks before referral, manual AK was performed at an outside institution for high astigmatism in a reportedly clear graft. Pre-AK best-corrected visual acuity (BCVA) and operative parameters—including arc length, optical zone, meridian, and intended incision depth—were unavailable. During the AK procedure a wound leak occurred at the superotemporal AK site and was immediately repaired with 2–3 sutures.

Corneal edema developed on the first postoperative day, and acute graft rejection was suspected. Topical corticosteroids were initiated, followed several days later by oral corticosteroids because the edema did not improve. Owing to the poor response and diagnostic uncertainty, she was referred to our clinic three weeks after AK as a case of presumed treatment-resistant graft rejection.

**Figure 1:**
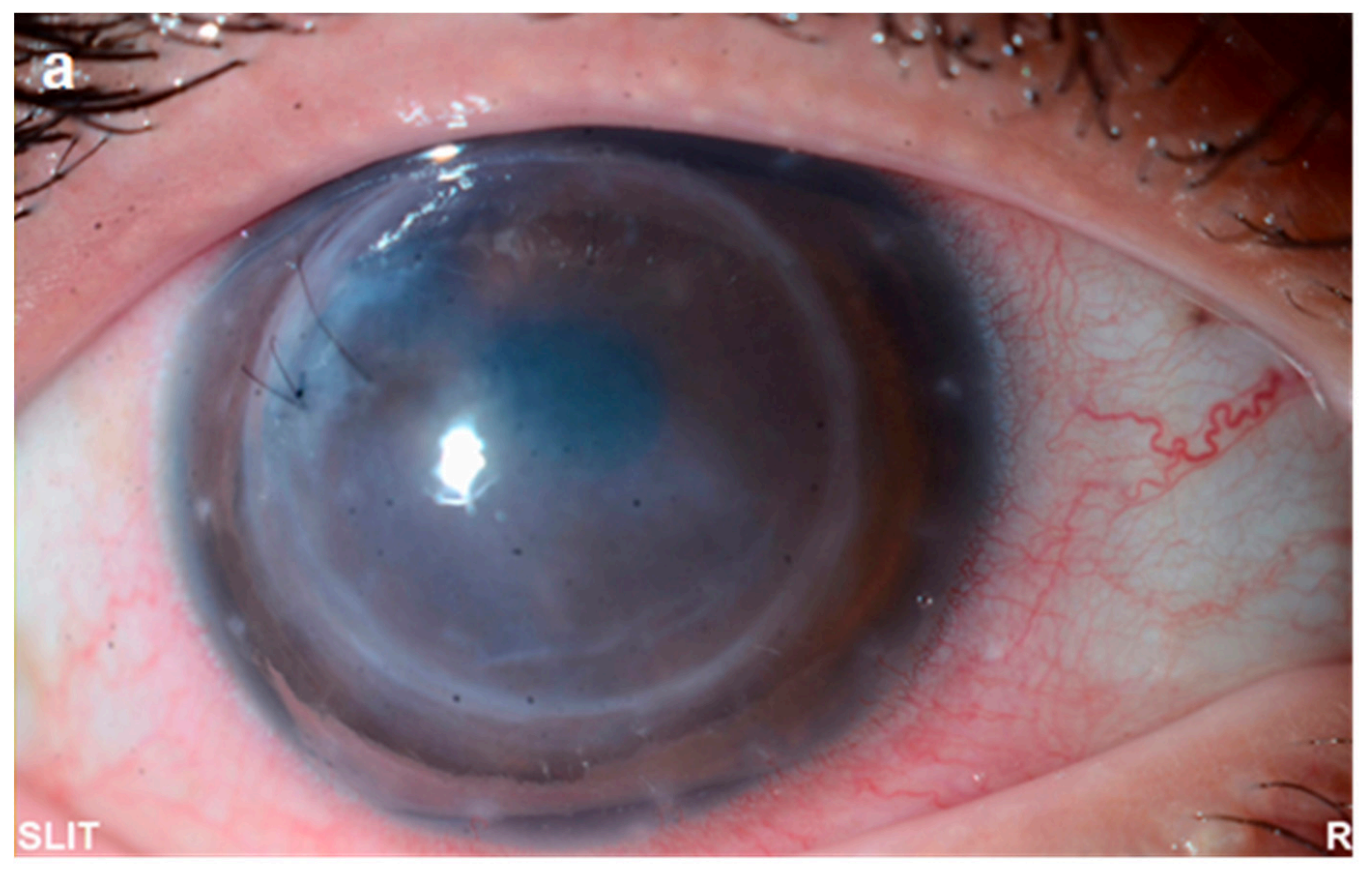

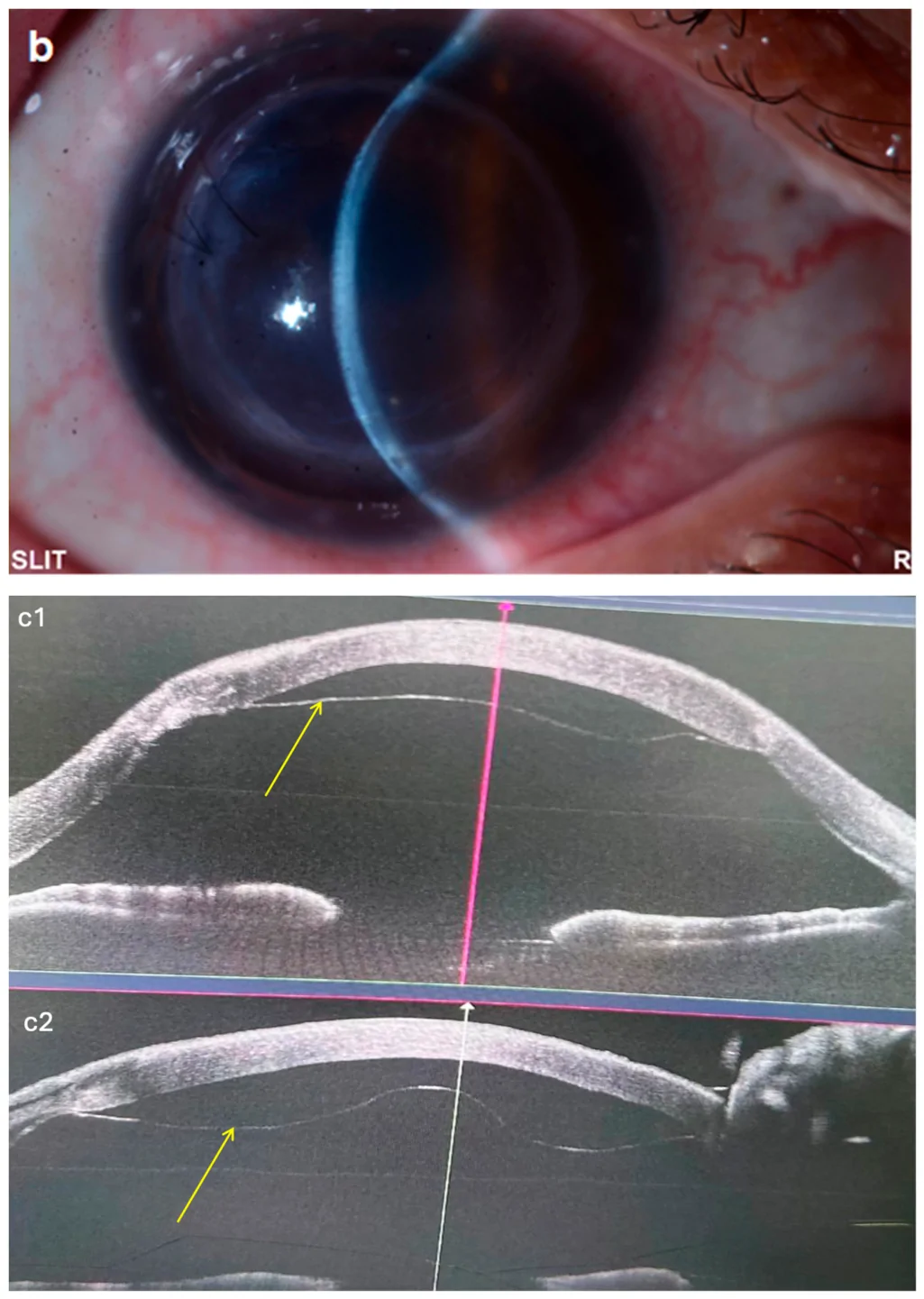
Clinical presentation at referral, before air descemetopexy. (**a**) Diffuse slit-lamp photograph demonstrating graft edema. (**b**) Slit-beam photograph demonstrating the detached Descemet membrane. Anterior segment optical coherence tomography demonstrating a near-total, nonplanar Descemet membrane detachment in the (**c1**) vertical and (**c2**) horizontal scans. Yellow arrows indicate the detached Descemet membrane.

At presentation, BCVA in the RE was hand motion (HM). Intraocular pressure (IOP) was documented as within normal limits in both eyes. Slit-lamp examination of the RE showed mild ciliary injection and diffuse graft edema. Further examination revealed visible DM folds and near-total DMD (Figure 1a,b). No keratic precipitates or rejection line was observed. The anterior chamber was deep, and the pupil was round and reactive. Fundus visualization was limited by corneal haze; after dilation, a mild-to-moderate cataract was noted. AS-OCT confirmed a near-total, nonplanar DMD in the RE (Figure 1(c1,c2)). B-scan ultrasonography showed no significant posterior-segment pathology.

After informed consent was obtained, a single intracameral air descemetopexy session was performed under sterile conditions with intracameral cefuroxime. Three sequential air injections were administered through an inferior paracentesis during the same session, with DM apposition reassessed after each injection. The procedure was concluded when full apposition was observed. A topical antibiotic and cyclopentolate three times daily were added to the existing treatment regimen.

**Figure 2:**
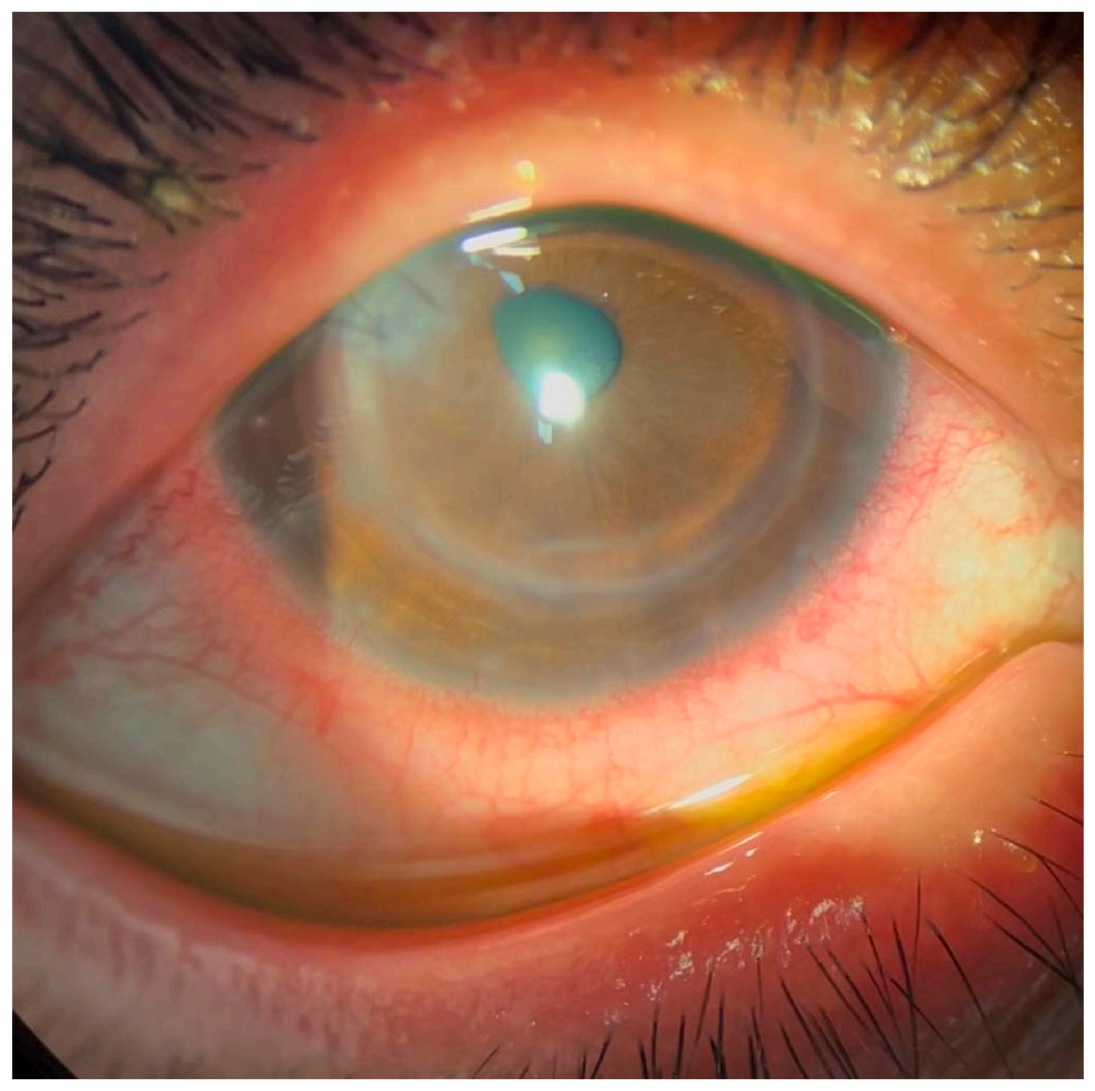
Slit-lamp photograph obtained after air descemetopexy, showing improved graft clarity compared with the presentation at referral.

**Figure 3:**
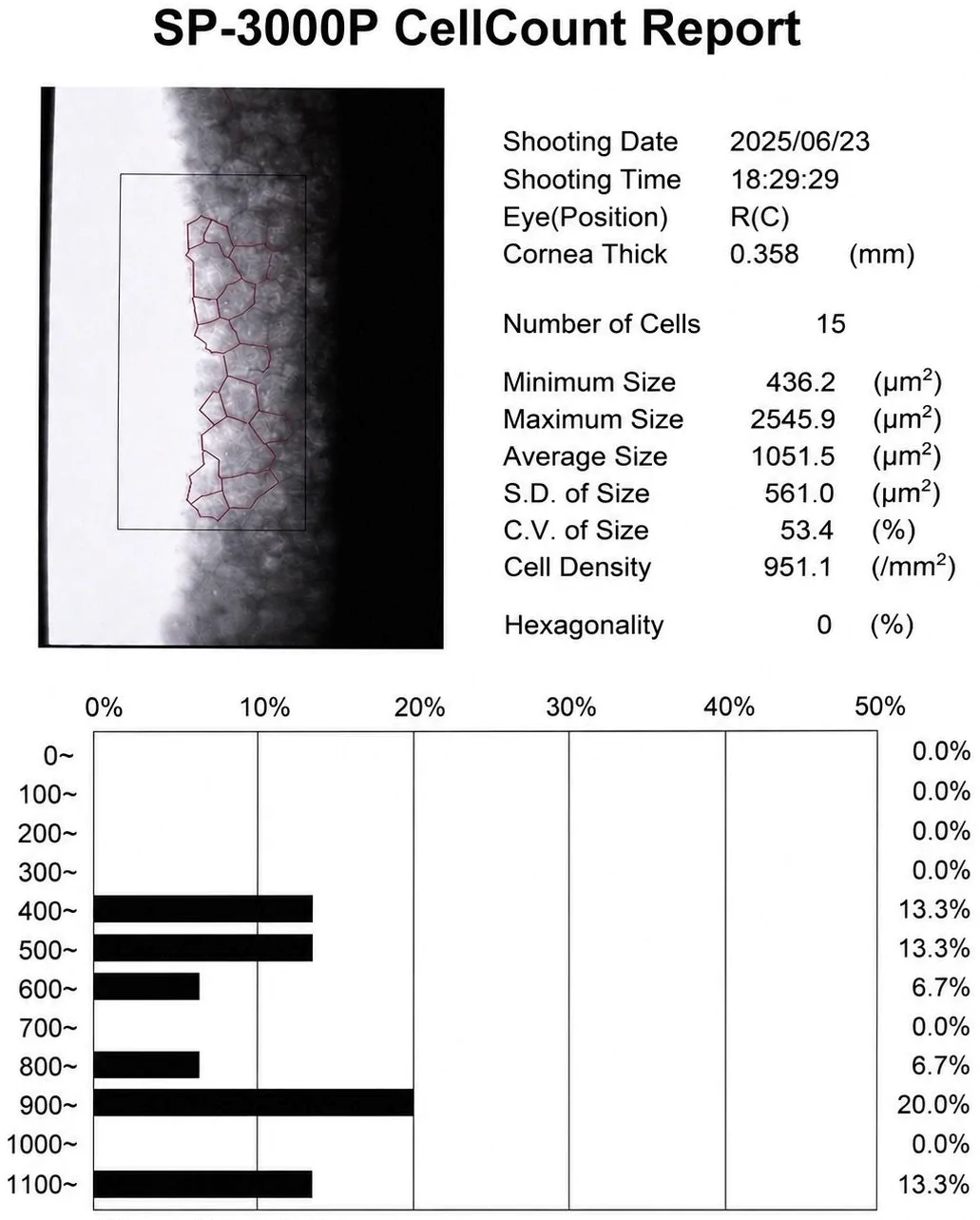
Post-treatment specular microscopy of the right corneal graft. The examination demonstrated an endothelial cell density of 951.1 cells/mm^2^ and a measured corneal thickness of 358 µm.

Following the air injections, slit-lamp examination demonstrated complete apposition of the DM with improvement in graft clarity (Figure 2). Post-treatment pachymetry showed a corneal thickness of 358 µm, and Specular microscopy (SP-3000P; Topcon Corporation, Tokyo, Japan) demonstrated an endothelial cell density of 951.1 cells/mm^2^ (Figure 3). At the 3-month follow-up, visual acuity had improved from HM at presentation to 20/200, and IOP was within normal limits. Refraction was not available. Post-treatment AS-OCT was not obtained. Further follow-up beyond 3 months was unavailable because the patient lived outside the city.

## 3. Discussion

The present case highlights DMD as an important differential diagnosis in patients presenting with graft edema after AK. The patient was initially treated for presumed acute graft rejection; however, the lack of clinical improvement prompted further evaluation, and AS-OCT subsequently demonstrated a near-total DMD. This emphasizes the value of early AS-OCT in patients with unexplained postoperative graft edema.

In a series of eyes that had undergone PKP for keratoconus, DMD occurred a median of 25 years after transplantation (range, 7–33 years), and several patients were initially treated for allograft rejection before AS-OCT established the diagnosis [13]. The 20-year interval after PKP falls within this reported range.

Determining the exact cause of DMD in the present case can be challenging. The DMD may have occurred due to accidental perforation during manual AK, mechanical stress from surgical instruments, or mechanical stress associated with the AK and subsequent wound suturing. This detachment could have led to corneal edema, further impairing visual acuity and requiring prompt intervention. AS-OCT played a key role in confirming the diagnosis. Mercer et al. similarly reported persistent edema after FSAK caused by an occult full-thickness wound complication identified using AS-OCT [14] in a review article that evaluated the efficacy and complications of FSAK for correction of astigmatism in patients who had previously undergone PKP. The article concluded that manual AK carries a higher risk of complications such as wound dehiscence, epithelial ingrowth, and full-thickness perforation compared with FSAK, which offers greater precision in incision depth and placement [15]. In the present case, the patient underwent manual AK, which may have contributed to the occurrence of DMD in the PKP graft.

Presentation of DMD versus acute graft rejection can be difficult because of overlapping clinical features such as graft edema and anterior chamber inflammation. Lack of keratic precipitates and presence of DM folds in this case pointed to DMD, which was confirmed by AS-OCT. AS-OCT is a valuable imaging tool for evaluating postoperative corneal edema and corneal graft pathology and it directly visualized a near-total separation of DM from the posterior stroma, which was not fully appreciated on clinical examination. Graft rejection in post-PKP eyes remains a serious sight-threatening complication, often triggered by inflammatory stimuli such as surgical trauma, infections, or delayed wound healing. The primary treatment is corticosteroid therapy. The development of graft edema in the present case shortly after AK and wound suturing initially raised clinical suspicion of acute graft rejection. However, the lack of response to corticosteroid therapy and the demonstration of a near-total DMD on AS-OCT supported a mechanical DMD rather than an immune-mediated graft rejection.

During one operative session, three sequential intracameral air injections resulted in full DM apposition on intraoperative assessment, with subsequent slit-lamp examination demonstrating improved graft clarity. At 3-month follow-up, visual acuity had improved from HM to 20/200, and IOP remained within normal limits. Post-treatment pachymetry showed a corneal thickness of 358 µm, and specular microscopy demonstrated an endothelial cell density of 951.1 cells/mm^2^. However, post-treatment AS-OCT and longer-term follow-up were unavailable; therefore, sustained anatomical and functional outcomes could not be assessed. No additional keratoplasty was performed during the documented 3-month follow-up period.

This clinical response is consistent with the existing literature supporting rebubbling as a safe and effective intervention for DMD, including in eyes with previous corneal surgery. A retrospective analysis of 112 cases of DMD reported a 71% reattachment rate following treatment with intracameral air injection alone. Of these, 15 patients needed further surgical procedures, achieving a 60% success rate in reattachment. One case with persistent corneal edema was eventually managed with endothelial keratoplasty. Complications observed included appositional angle closure in 18% of cases, pupillary block in 2.1%, and uveitis in 2.7% [16]. These findings are consistent with the clinically observed reattachment following single-session air descemetopexy in the reported patient, despite the added complexity of a previous PKP graft.

According to the literature, DMD is a case-based strategy that can be treated conservatively with medication or surgically. Hyperosmotic agents and topical steroids are involved in conservative therapy. However, simple pneumodescemetopexy has shown more success, especially for scrolling, severe, and visually impaired DMDs, and authors now advocate for early surgical repair [17]. Air or gas tamponade is considered the first-line treatment for DMD, with a good success rate for tiny and planar detachments. Particularly in post-PKP corneas, nonplanar or complete detachments may necessitate several injections and have a higher failure rate [3]. The present case demonstrates that an extensive DMD may achieve clinical reattachment following air descemetopexy, although multiple sequential air injections were required during the same operative session.

There are some similar reported cases of a PKP graft rejection following corneal procedures; for instance, a 46-year-old woman with previous PKP for keratoconus, about 20 years prior developed corneal edema, deep Descemet folds, and keratic precipitates consistent with acute immune graft rejection after a Photorefractive Keratectomy (PRK) performed to correct high residual astigmatism [18].

The reported case highlights the importance of considering DMD as a potential cause of graft edema after AK even if there is no obvious intraoperative perforation. Early recognition of this possibility may help avoid delays in diagnosis and ensure appropriate management.

Further research is needed to clarify whether some cases of postoperative corneal edema following AK represent self-resolving DMD rather than acute graft rejection.

### Limitation

As a single case report, the findings cannot be generalized to all patients with DMD following AK in eyes with previous PKP. The AK was performed at an outside institution; therefore, detailed information regarding the indication, intraoperative findings, technique, and perioperative management was unavailable. Pre-AK BCVA was also unavailable, as this measurement was not included in the referral documentation. Follow-up was limited to 3 months, precluding assessment of long-term visual outcomes and graft survival. Post-treatment AS-OCT was not obtained; therefore, DM reattachment was assessed clinically by slit-lamp examination, supported by post-treatment pachymetry and specular microscopy.

## 4. Conclusions

This case represents the first reported instance of DMD following AK in the setting of a previous PKP. It underscores the importance of including DMD in the differential diagnosis of postoperative corneal edema, even in atypical surgical contexts. Prompt recognition with AS-OCT and appropriate management with air descemetopexy resulted in Descemet membrane apposition, improved graft clarity, and improvement in visual acuity from HM to 20/200 at 3 months.

## Data Availability

The original data presented in this study are available on reasonable request from the corresponding author. The data are not publicly available due to privacy concerns.

## Abbreviations

The following abbreviations are used in this manuscript:

AK	Arcuate keratotomy
AS-OCT	Anterior segment optical coherence tomography
BCVA	Best-corrected visual acuity
DM	Descemet membrane
DMD	Descemet membrane detachment
FSAK	Femtosecond laser-assisted arcuate keratotomy
HM	Hand motion
IOP	Intraocular pressure
PKP	Penetrating keratoplasty
PRK	Photorefractive keratectomy
RE	Right Eye

## References

[1] Beniwal A., Vanathi M., Venugopal A., Chaurasia S., Tandon R. (2024). Descemet’s membrane detachment: An updated comprehensive review of etiopathogenesis, diagnosis, and management. Indian J. Ophthalmol..

[2] Moramarco A., Iannetta D., Cimino L., Romano V., Gardini L., Fontana L. (2023). Case Report: “Spontaneous Descemet Membrane Detachment”. J. Clin. Med..

[3] Chow V.W.S., Agarwal T., Vajpayee R.B., Jhanji V. (2013). Update on diagnosis and management of Descemet’s membrane detachment. Curr. Opin. Ophthalmol..

[4] Sharma N., Gupta S., Maharana P., Shanmugam P., Nagpal R., Vajpayee R.B. (2015). Anterior Segment Optical Coherence Tomography–Guided Management Algorithm for Descemet Membrane Detachment After Intraocular Surgery. Cornea.

[5] Trindade B.L.C., Attanasio De Rezende R., Bisol T., Rapuano C.J. (2023). Late Descemet membrane detachment after uneventful cataract surgery. Am. J. Ophthalmol. Case Rep..

[6] Ruggeri F., Rullo D., Maugliani E., Trotta N., Ciancimino C., Di Pippo M., Guglielmelli F., Abdolrahimzadeh S. (2025). The role of anterior segment optical coherence tomography in post-cataract surgery Descemet membrane de-tachment. Int. Ophthalmol..

[7] Guccione L., Mosca L., Scartozzi L., Crincoli E., Fasciani R., Caporossi T., Rizzo S., Lo Giudice G. (2022). Correction of Refractive Errors after Corneal Transplantation. Vision Correction and Eye Surgery [Internet].

[8] Deshmukh R., Nair S., Vaddavalli P.K., Agrawal T., Rapuano C.J., Beltz J., Vajpayee R.B. (2022). Post-penetrating keratoplasty astigmatism. Surv. Ophthalmol..

[9] Hoffart L., Touzeau O., Borderie V., Laroche L. (2007). Mechanized astigmatic arcuate keratotomy with the Hanna arcitome for astigmatism after keratoplasty. J. Cataract Refract. Surg..

[10] St. Clair R.M., Sharma A., Huang D., Yu F., Goldich Y., Rootman D., Yoo S., Cabot F., Jun J., Zhang L. (2016). Development of a nomogram for femtosecond laser astigmatic keratotomy for astigmatism after keratoplasty. J. Cataract Refract. Surg..

[11] Fadlallah A., Mehanna C., Saragoussi J.-J., Chelala E., Amari B., Legeais J.-M. (2015). Safety and efficacy of femtosecond laser–assisted arcuate keratotomy to treat irregular astigmatism after pene-trating keratoplasty. J. Cataract Refract. Surg..

[12] Kumar N.L., Kaiserman I., Shehadeh-Mashor R., Sansanayudh W., Ritenour R., Rootman D.S. (2010). IntraLase-Enabled Astigmatic Keratotomy for Post-Keratoplasty Astigmatism: On-Axis Vector Analysis. Ophthalmology.

[13] Kit V., Kriman J., Vasquez-Perez A., Muthusamy K., Thaung C., Tuft S. (2020). Descemet Membrane Detachment After Penetrating Keratoplasty for Keratoconus. Cornea.

[14] Mercer R., Loomba A., Goel S. (2024). Penetrating femtosecond arcuate keratotomy presenting with corneal edema. Indian J. Ophthalmol.—Case Rep..

[15] Chang J.S.M. (2018). Femtosecond laser-assisted astigmatic keratotomy: A review. Eye Vis..

[16] Odayappan A., Shivananda N., Ramakrishnan S., Krishnan T., Nachiappan S., Krishnamurthy S. (2018). A retrospective study on the incidence of post-cataract surgery Descemet’s membrane detachment and out-come of air descemetopexy. Br. J. Ophthalmol..

[17] Singhal D., Sahay P., Goel S., Asif M.I., Maharana P.K., Sharma N. (2020). Descemet membrane detachment. Surv. Ophthalmol..

[18] Spadea L., Giannico M.I., Armentano M., Alisi L., Pistella S. (2021). Acute corneal graft rejection following photorefractive keratectomy for post-penetrating keratoplasty high astigmatism. Int. J. Ophthalmol..

